# The Impact of LiDAR Configuration on Goal-Based Navigation within a Deep Reinforcement Learning Framework

**DOI:** 10.3390/s23249732

**Published:** 2023-12-09

**Authors:** Kabirat Bolanle Olayemi, Mien Van, Sean McLoone, Stephen McIlvanna, Yuzhu Sun, Jack Close, Nhat Minh Nguyen

**Affiliations:** School of Electronics, Electrical Engineering and Computer Science, Queen’s University Belfast, Belfast BT9 5AG, UK; kolayemi01@qub.ac.uk (K.B.O.); s.mcloone@qub.ac.uk (S.M.); smcilvanna01@qub.ac.uk (S.M.); ysun32@qub.ac.uk (Y.S.); jclose06@qub.ac.uk (J.C.); nnhat01@qub.ac.uk (N.M.N.)

**Keywords:** reinforcement learning, deep-reinforcement learning, collision avoidance, husky, gazebo, LiDAR, beam, field of view

## Abstract

Over the years, deep reinforcement learning (DRL) has shown great potential in mapless autonomous robot navigation and path planning. These DRL methods rely on robots equipped with different light detection and range (LiDAR) sensors with a wide field of view (FOV) configuration to perceive their environment. These types of LiDAR sensors are expensive and are not suitable for small-scale applications. In this paper, we address the performance effect of the LiDAR sensor configuration in DRL models. Our focus is on avoiding static obstacles ahead. We propose a novel approach that determines an initial FOV by calculating an angle of view using the sensor’s width and the minimum safe distance required between the robot and the obstacle. The beams returned within the FOV, the robot’s velocities, the robot’s orientation to the goal point, and the distance to the goal point are used as the input state to generate new velocity values as the output action of the DRL. The cost function of collision avoidance and path planning is defined as the reward of the DRL model. To verify the performance of the proposed method, we adjusted the proposed FOV by ±10° giving a narrower and wider FOV. These new FOVs are trained to obtain collision avoidance and path planning DRL models to validate the proposed method. Our experimental setup shows that the LiDAR configuration with the computed angle of view as its FOV performs best with a success rate of 98% and a lower time complexity of 0.25 m/s. Additionally, using a Husky Robot, we demonstrate the model’s good performance and applicability in the real world.

## 1. Introduction

Unmanned autonomous vehicles have been studied for decades and have been used increasingly for many real-life applications, such as search and rescue operations, military supplies delivery, transport of agricultural products or materials, delivery of materials to different sections of a warehouse, delivery of customer orders in restaurants, and delivery of clinical supplies within the hospital [1]. To implement these applications, LiDAR sensors play a crucial role in improving the situational awareness and navigation capabilities of robots. For example, self-driving cars and delivery robots use LiDAR sensors to aid autonomous driving, path planning, and collision avoidance. The LiDAR sensor helps the vehicle understand the surrounding environment, detect lane boundaries, and identify other vehicles, pedestrians, or obstacles. However, research has shown that the main challenges faced by unmanned autonomous vehicles are to accurately perceive their environment and to learn and develop a policy for safe and autonomous navigation [2]. Therefore, despite the huge amount of research in the field, existing systems have not yet achieved full autonomy in terms of collision avoidance, risk awareness, and recovery.

Before the use of LiDAR information became popular, visual simultaneous localization and mapping (vSLAM) was often used to perceive environment dynamics. In vSLAM, an operator measures and generates a map showing the locations, landmarks, and the guided path to the goal in the environment. However, such visual mapping systems have two major limitations: (1) they cannot reliably identify obstacles in low light conditions or when dealing with repetitive patterns, and (2) processing visual data can be computationally intensive [3]. Unlike vSLAM, LiDAR uses eye-safe laser beams to capture the surrounding environment in 2D or 3D providing computing systems with an accurate representation of its environment that prompts its use by many automobile companies such as Volkswagen, Volvo, and Hyundai for autonomous driving, object detection, mapping, and localization [4].

**Figure 1:**
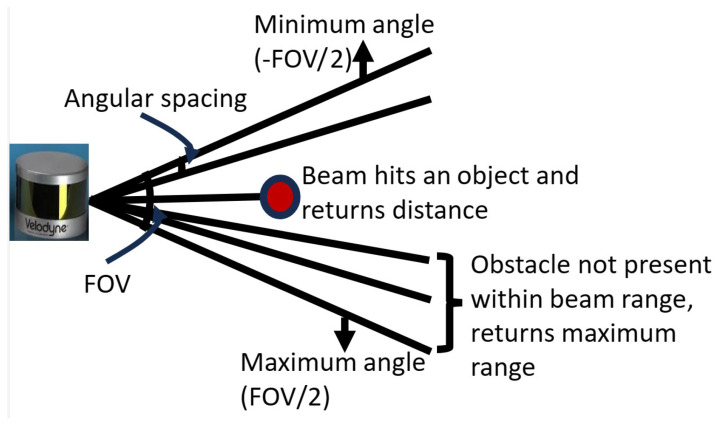
LiDAR sensor parameter definition.

LiDAR sensors can be categorized as mechanical, semi-solid, or solid-state based on their scanning methods. Mechanical scanning LiDARs use a rotating mirror or prism to direct the laser beam over 360° using a motor. This type of LiDAR design is very expensive and large; hence, it is not suitable for large-scale use. In semi-solid-state LiDAR sensors, the mechanical rotating parts are made smaller and hidden within the shell of the LiDAR sensor, making the rotation invisible from its appearance. Solid-state LiDAR sensors do not have moving parts. They use electronic components to steer the laser beams, thereby reducing the cost of production and improving efficiency and reliability. They are also more durable and compact, making them suitable for automotive applications. The general characteristics of a LiDAR sensor are defined by the angular range $(angle_{min}$ and $angle_{max})$, resolution, sampling rate, and range, as shown in Figure 1. The angular range determines the FOV covered by the LiDAR sensor, that is, the extent of the environment that the LiDAR can capture in terms of angles. The resolution parameter determines the angular spacing between individual beams within the specified FOV. A small resolution value allows more beams within the same FOV and results in increased coverage and potentially higher-resolution data. The sampling rate defines the number of beam pulses that the LiDAR sensor will emit, while the range determines how far the beam can travel. These parameters allow the design of different models of LiDAR sensors.

The 2D LiDAR sensors emit laser beams in a single plane (horizontal or vertical) to create a 2D representation of the environment. It measures the time it takes for the laser beam to return after hitting an object, allowing it to calculate distances and generate a 2D point cloud. In contrast to the 2D LiDAR sensor, the 3D LiDAR sensor emits a much larger number of laser beams in multiple directions (usually both horizontally and vertically) to create a volumetric representation of the environment. Sensors with a very high number of beams come with a higher price tag and are, therefore, not cost effective for smaller applications.

Due to the ability of LiDAR sensors to provide a real-time representation of the robot’s environment, they have increasingly been exploited to generate point clouds of the environment for training DRL models for autonomous self-driving vehicles [5,6,7]. For cost effectiveness, researchers typically use 2D LiDAR sensors for small-height robots [8,9,10]. In addition, different researchers randomly select different LiDAR sensors with different beam densities and FOV configurations. Since the DRL model makes decisions based on the information received from the LiDAR sensor, it is important to understand the beam density and FOV required to develop an appropriate learning model. This leads us to ask if a large beam size is required to navigate a static environment.

**Figure 2:**
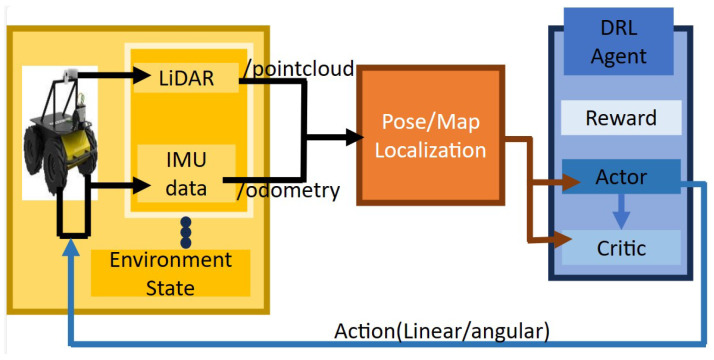
The robot’s x,y position is obtained from the robot odometry while the distance between the robot and the obstacle are calculated from the LiDAR information, which is passed to the neural network model as input. After training, a navigation policy is produced to command the robot’s velocities at each time step.

Hence, in this paper, we use a novel DRL-based approach to explore the performance of an autonomous ground vehicle driving through its environment to a goal point based on the information obtained from different LiDAR sensor configurations. Without prior knowledge of the environment, a point of interest is extracted from the robot’s present surroundings and evaluated, and then a routing point is selected to guide the DRL policy formation. Our approach calculates an initial FOV to generate a learning model referred to as model 2 in this work, after which two different FOVs are generated to achieve a narrower and wider FOV for comparison. The emitted beams are used to estimate the distance from the robot to the obstacle ahead to obtain a path from the initial pose of the robot to the goal point. The input to the neural network model is the linear and angular velocity of the robot, the orientation of the robot, the current distance between the robot and its goal, and the LiDAR information, while the output is the next velocity values of the robot (linear and angular) for the next time step, as shown in Figure 2. Despite the common use of DRL to generate an obstacle avoidance control policy, the overall contribution of this work is as follows.

1. We design a DRL control policy based on goal-based exploration.
2. We explore the effect of the LiDAR beam and FOV on the performance of the DRL model by learning the appropriate FOV and beam density suitable for a static environment. This is essential when the application needs a low-resolution LiDAR sensor.
3. We demonstrate the performance of our model in a simulated environment to test the effect of different LiDAR sensor configurations on collision avoidance.
4. We demonstrate our control policy on a Husky A200 robot by Clear Robotics using environment dynamics different from those used for training.

The rest of the paper is organized as follows. In Section 2, we describe the work related to the study. Section 3 introduces the problem formulation, the elements that constitute the environment, and the methodology used in the paper. Section 4 describes the training environment (gazebo) and the training process. Experimental results and performance are discussed in Section 5. Section 6 describes the limitations of the proposed method and future work. Finally, in Section 7, we conclude the paper.

## 2. Related Work

Collision avoidance and path planning problems have been investigated and solved using many techniques, such as RRT, A, A*, RRT*, and decision trees [11,12,13,14]. These techniques are mostly suitable for applications where the environment state is known and not changing. In a bid to offer a better collision avoidance solution, researchers introduced map localization and position-based methods. In map localization and positioning, cameras are used to capture the state of the environment and detect obstacles and their sizes to determine the path to follow [15]. This usually follows the process of mapping, localization, and planning. In this scenario, as with A, RRT, and related approaches, the environment needs to be known beforehand to design a policy for path planning; hence, it is not best suited for a dynamic environment [16,17,18]. Furthermore, the environment used to develop the model can change over time, and maintaining and updating the models ensures that it can adapt to changes and continue to navigate safely, which is costly, time-consuming, and requires knowledge and experience.

In recent years, the use of reinforcement learning and DRL has increased significantly due to its excellent environmental awareness and decision control performance [19]. The ATAri 2600 [20] and AlphaGo [21] developed by DeepMind are two of the early success stories of RL. In mobile robots, the use of RL/DRL to directly map the state of the environment to the control signal for a dynamic path planning solution remains a challenge. Kasun et al. [22] investigated robot navigation in an uneven outdoor environment using a fully trained DRL network to obtain a cost map to perform the navigation task. The network has prior knowledge of the environment by accepting elevation maps of the environment, the robot poses, and the goal axis as input. Xue et al. [23] and Ruan et al. [24] investigated the use of a double-deep Q-network (DDQN). The size and position of the obstacle and the target position are taken as input to the network, and the robot’s velocity values are output. In [25], a deep deterministic policy gradient (DDPG) algorithm was used to select a control policy for hybrid unmanned aerial underwater vehicles using the robot’s state, LiDAR measurements, and distance to the goal point. A goal-orientated approach to obstacle avoidance was implemented by [26,27]. Their work was based on processing a large amount of depth image information using DRL to reach its goal while avoiding obstacles in a continuous or unknown environment. In another work, Choi et al. [28] proposed the integration of both path planning and reinforcement learning methods to predict the next movement of an obstacle using the calculated distance from the LiDAR information. Wang et al. [29] implemented the curriculum learning of a DRL robot to navigate among movable obstacles. Rather than collecting human demonstrations as in [30], they introduced the use of prior knowledge.

Most collision avoidance models developed using DRL obtain the state of the environment through LiDAR information. When training the learning network, many researchers have used different FOVs (90°, 180°, 270°, or 360°), and the number of LiDAR beams (10–60) has been used by many researchers, which directly impacts the computational complexity of the network [31,32]. Tai et al. [33] developed a navigation learning model in a simulated environment using a 10-dimensional laser beam as one input to the model. Han et al. [34] used the fusion of RGB images from a camera and 2D LiDAR sensor data as input to a DRL network of self-state attention to investigate the effect of using 2D LiDAR on a tall robot. In their work, the training environment is captured and processed before passing it to the training network. Xie et al. [35] applied a proportional-integral-derivative (PID) controller to improve the training rate of a convolutional neural network that takes 512 stacked laser beams as input. In [36], a reinforcement learning method is developed that automatically learns the best number of beams required based on the application. Their work was tested on object detection and shows that the appropriate beam configuration improves the performance of the LiDAR application. Zhang et al. [37] developed a neural network for safe navigation based on different LiDAR sensor configurations (FOV, number of LiDAR sensors mounted and LiDAR orientation). Their work shows that the models with a LiDAR sensor with an FOV of 240° in all scenarios perform better than all other FOVs used. Another work by [38] chooses an FOV with a minimum angle of 13.4° and a maximum angle of 11.5° to train a DRL model for robot navigation. Their approach was able to navigate safely with the limited FOV. Jinyoung et al. [39] investigated the performance of a narrow FOV LiDAR in robot navigation. They developed a navigation model using long-short-term Memory (LSTM), a type of recurrent neural network with a local-map critic (LMC). However, these researchers did not provide details of the criteria used in the selection of these FOVs.

A LiDAR sensor emits light beams to its surroundings, which in turn bounce off surrounding objects back to the sensor. The beam that takes the shortest time to return to the sensor is used to calculate the shortest distance to an impending obstacle, which is further used to control the robot velocity values while training a neural network during navigation. Therefore, it is important to investigate the effect of the LiDAR sensor configuration required to train a DRL based on the required application. Does the DRL learning algorithm require 360°, 270°, 90°, or other view of the environment to be effective? To this end, we propose a method of estimating the required FOV based on the width of the sensor and the obstacle in view. Table 1 summarizes the differences between our approach and the existing literature.

## 3. Problem Formulation

In this investigation, we are considering the transfer of a mobile robot from a starting point to a known target position while avoiding obstacles. To achieve a successful autonomous exploration, it is required that the mobile robot avoids colliding with obstacles along its path, while at the same time getting to its target in the shortest distance and travel time. To formulate the problem, the properties of the robot, the dynamics of the environment, and the reinforcement learning model are discussed in this section.

### 3.1. Simulation Environment

**Figure 3:**
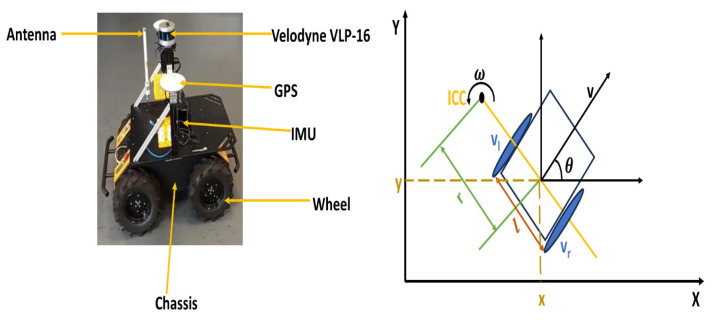
Husky A200 UGV (**left side**) is a differential drive wheeled unmanned ground vehicle designed for robotic research. The (**right side**) illustrate the differential drive for mobile robot.

*Mobile Robot Dynamics:* For our experiment, we will use a simulation of Husky A200 UGV developed by Clearpath Robotics, Inc., Ontario, Canada. This is a non-holonomic, differential-drive mobile robot, as shown in Figure 3, which allows the control of its linear (forward or backward) and angular (left or right rotation) velocities. The relationship between the instantaneous center of curvature (ICC) along the left velocity of the ground wheel $\left(v_{l}\right)$ and the right velocity $\left(v_{r}\right)$, expressed numerically in radians per second (rad/s), is defined as [40,41,42,43]:

(1) $$r=\frac{l(v_{r}+v_{l})}{2(v_{r}-v_{l})}$$

(2) $$v=\frac{\left(v_{r}+v_{l}\right)}{2}$$

(3) $$w=\frac{\left(v_{r}-v_{l}\right)}{l}\;$$

where *r* is the radius of the driving wheel, *v* is the linear velocity expressed in meters per second (m/s), *w* is the angular velocity expressed in (rad/s), and *l* is the distance between the wheelbase. The kinematic model $\left(\dot{q}\right)$ of the differential drive of the mobile robot is thus given as:

(4) $$\dot{q}=\left[\begin{array}{c} \dot{x}\\ \dot{y}\\ \dot{\theta } \end{array}\right]=\left[\begin{array}{c} vcos(\theta )\\ vsin(\theta )\\ w \end{array}\right]$$

In the model presented, the location of the robot is defined by its Cartesian position $\left(\dot{x},\dot{y}\right)$ and the orientation $\left(\dot{\theta }\right)$. Since the maximum speed of the robot is 1 m/s, the linear speed is set within the range of [0,1] and the angular rotation within [−1,1].

**Figure 4:**
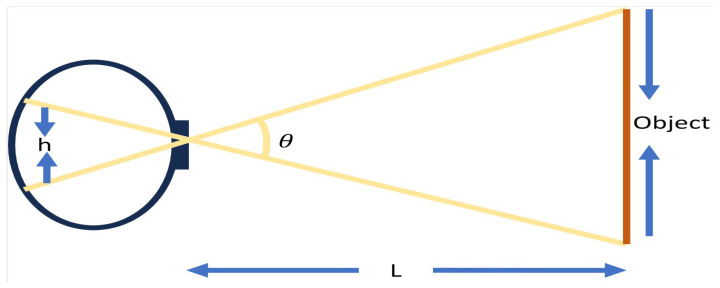
Illustration of viewing angle of an object. The viewing angle is obtained on the basis of the sensor width (h) and distance to the obstacle (L).

*LiDAR Sensor Model:* For our investigation, we consider map-less robot navigation of its surroundings. First, we consider the required angle of view suspended by an object as depicted in Figure 4. Denoting the width of the sensor used as *h* in millimeters, the minimum distance between the obstacle and the robot as *L* in millimeters, the angle of view θ is calculated as:

(5) $$\theta =2*\arctan\frac{h}{2*L}$$

For our experiment, the distance between the LiDAR sensor mounted on the robot and the obstacle is set at a minimum of 100 mm apart while the width of the sensor in use is 103 mm. From the calculated angle of view, we obtain our proposed LiDAR FOV $\theta_{y}$. For verification, we obtain a narrower FOV $\theta_{x}$ and a wider FOV $\theta_{z}$. These three FOVs are used to generate three navigation models. For each of the FOVs, there are different beam densities, that is, a different number of beams ($n_{x}$, $n_{y}$, and $n_{z}$) distributed uniformly throughout the FOV. The angular spacing Θ between the beams is given as:

(6) $$\Theta =\frac{\theta }{n_{x,y,z}-1}$$

**Figure 5:**
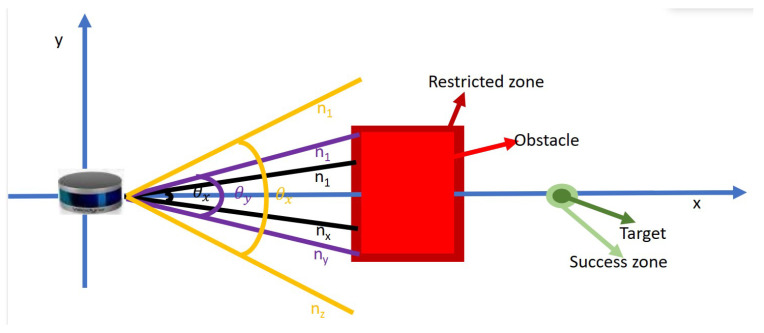
Illustration of a LiDAR sensor with different FOV and beam densities. FOV $\theta_{x},\theta_{y},\theta_{z}$ has a beam density of $n_{x},n_{y},n_{z}$ respectively.

The maximum range $n_{max}$ each beam can travel is set to 10 m, while the resolution is set to 1. These parameters are used to define three different LiDAR sensor configurations. For each model configuration, an array of point cloud beams from the LiDAR sensor is used to perceive the robot’s surroundings, enabling it to avoid obstacles and reach its goal point, as shown in Figure 5. If no obstacle is detected, the laser returns $n_{max}$ as the free distance ahead or ranging values of *n* picking the least value as the distance to the first obstacle it encounters.

**Figure 6:**
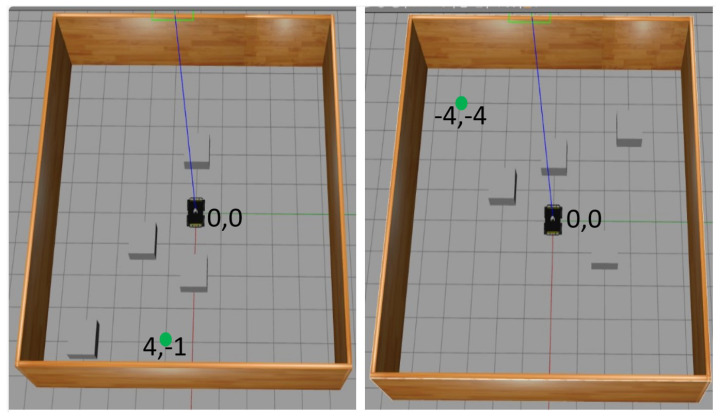
Sample of the simulated environment used for training. The robot position (x,y) is (0,0) in both environments. Goal points are (4,−1) and (−4,−4) for the left and right environments, respectively.

Obstacle Environment: For this experiment, a $10\times 10\;m^{2}$ square wall was used to restrain the robot from going out of the experiment space. At the beginning of each episode, the target point is also changed, and the positions of all four obstacles of the same shape and size are randomly placed in the experiment space, as shown in Figure 6. The purpose is to randomly initialize the training data set.

### 3.2. Action Space and State Representation

Developing a reinforcement learning-based unmanned robot is based on four components: (1) the robot model and its environment, (2) the state and action space, (3) the policy and reward function, and (4) the sensing, motion, and communication units [44,45]. In this paper, navigation in an unknown environment is based on using the current information received by the LiDAR at each time step to control the linear or angular velocity of the vehicle. Given the initial $x_{i},y_{i}$ and final coordinates $x_{f},y_{f}$ of the robot, the probability of transitioning from one state to another $P(s^{\prime }|s)$ depends on the distance between the robot and the target point $d_{t}^{g}$, the orientation of the robot to the goal point $\omega_{t}^{g}$, the previous linear velocity $v_{t}$, the angular velocity $w_{t}$, and an array *N* of LiDAR beams *n* relative to the distance between the obstacle and the robot at each time step *t*:

(7) $$s_{t}=\left[n,d_{t}^{g},\omega_{t}^{g},v_{t},w_{t}\right]$$

The value of *n* depends on the LiDAR configuration used, that is, $n\in (n_{x},n_{y},n_{z})$. The next action space in the time step *t* consists of the linear and angular velocity $A=[v_{t}^{\prime },w_{t}^{\prime }]$ obtained from the policy distribution $\pi (s_{t})$.

### 3.3. Deep-Reinforcement Learning

Reinforcement learning is a process where an agent (controller) is reinforced with rewards or penalties based on its interaction with its environment. It learns to take the appropriate action to maximize the reward in the environment. Formally, every reinforcement learning problem is formulated as a Markov decision process (MDP) [46]. An MDP is represented as a five-tuple 〈S,A,P,R,γ〉, where *S* is a set of states the agent can be in, *A* is a finite set of actions the agent can take, *P* is a state transition probability matrix, $P_{ss{}^{\prime }}^{a}=P\left[S_{t+1}=s^{\prime }|S_{t}=s,A_{t}=a\right]$, *R* is a reward function, $R_{s}^{a}=E\left[R_{t+1}|S_{t}=s,A_{t}=a\right]$ following a discount factor γ∈[0,1]. This shows that the probability of transiting between state $S_{t}$ and $S_{t+1}$ depends on the action $A_{t}$. At each time step *t*, the agent chooses an action based on a policy $\theta_{\pi }\left(a|s\right)$.

In cases where the dynamics of the environment are unknown, Q-learning is the widely used method to solve MDPs. Q-learning is a model-free, off-policy RL algorithm that learns directly from its experience of interacting with the environment. The algorithm aims to learn an optimal action-value function (Q-function), which assigns the expected cumulative reward the agent can receive by taking a specific action in each given state to each action–state pair [47]. The Q-table is represented as a 2D matrix, where the rows represent states, and the columns represent actions.

(8) $$Q\left(s,a\right)=\;Q\left(s,a\right)+\alpha^{*}\left(r+\gamma^{*}max\left[Q\left(s^{\prime },a^{\prime }\right)\right]-Q\left(s,a\right)\right)$$

**Figure 7:**
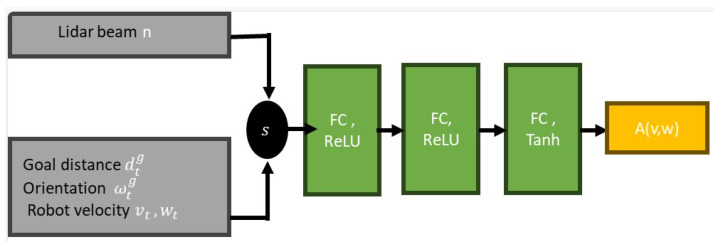
The actor network.

In DRL, the actor–critic approach is applied to approximate the Q function [48]. The actor, which contains the policy-based algorithm modeled as a neural network with parameters $\pi_{\theta }$, selects the action of the agent, while the critic or the value-based algorithm $V^{\pi }\left(a|s\right)$ evaluates the actions and suggests the best action-value function $A^{\pi }\left(a|s\right)$. The critic also helps to account for the discounted sum of future rewards. In this work, a twin delay deep deterministic policy gradient (TD3) actor–critic network is used to train the control policy. As shown in Figure 7, the actor network is composed of the observation state *s* as its input value. The first hidden layer is a fully connected layer (FC) with 800 units with rectified linear unit (ReLU) activation functions used to refine the representation of the state. The second hidden FC layer with 600 units also uses ReLU activation functions to further refine the representation of the state. The last FC layer has two units and represents the output action dimension. A tanh activation function is used to squash the output to be within the range [−1, 1].

**Figure 8:**
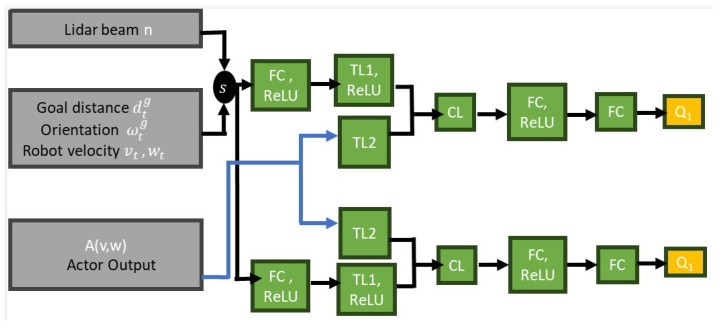
The critic network.

For the critic network, two networks are used to evaluate Q(s,a). As shown in Figure 8, the two networks have a similar architecture. The first hidden layer has 800 units. It takes the state representation as input and applies the ReLU activation function to introduce non-linearity to the network. The output of this layer is passed to the first transformation layer (TL1). The TL1 has 800 units and uses ReLU activation to further introduce non-linearity to the network. The output from the actor network is passed as input to the second transformation layer (TL2), which transforms the action to match the dimensionality of the state representation without introducing non-linearity to the network. The combined layer (CL) concatenates the state and the transformed action, creating a vector of features. The CL output is passed to an FC layer with 800 units and applies ReLU activation. The output from the FC layer consists of a single unit representing the estimated Q-value.

### 3.4. Reward Function

Based on the action of the robot in an environment, we create our reward function to guide DRL training to encourage desirable actions while traveling between states. A positive or negative reward is given based on the action and state space. Considering the navigation space of mobile robots in this research, the reward is based on the robot’s ability to navigate to its destination while avoiding obstacles. To define the reward function, the following reward variables were considered:

1. *Collision Penalty:* A 0.3 m radius zone is placed around the obstacle called the restricted zone. It is considered that a collision has occurred if the robot enters the restricted zone. The collision penalty is defined as:
  (9) $$r_{c}=\left\{\begin{array}{cc} -(100-\frac{d_{o}}{d_{ot}}), & \mathrm{if}d_{o}<=d_{ot}\\ 0, & \mathrm{otherwise}. \end{array}\right.$$
  where $d_{o}$ is the distance between the robot and the obstacle, and $d_{ot}$ is the distance threshold from the obstacle.
2. *Goal Reward:* Like the restricted zone, a 0.2 m radius surrounding the goal point is referred to as the success zone. If the robot enters the success zone, it is considered that the robot has reached its goal. Equation (10) shows the calculated goal reward.
  (10) $$r_{g}=\left\{\begin{array}{cc} 100, & \mathrm{if}d_{g}<=d_{gt}\\ 0, & \mathrm{otherwise}. \end{array}\right.$$
  where $d_{g}$ is the distance between the robot and the goal point and $d_{gt}$ is the goal threshold to the target point.
3. *Distance Penalty:* This is the penalty obtained based on the distance between the robot and the target point $d_{g}$ relative to the initial distance to the target point $d_{i}$. If the robot is close to the goal point, it receives a small penalty, while if the distance is large, it receives a large penalty.
  (11) $$r_{d}=-\frac{d_{g}}{d_{i}}$$
4. *Heading Penalty:* To ensure that the robot heads toward the goal point, a penalty is placed on the robot’s orientation. Given the robot’s orientation $\omega_{r}$, and the goal point orientation $\omega_{g}$, the heading penalty is calculated as:
  (12) $$r_{h}=\left\{\begin{array}{ll} -\left(\frac{|\omega_{g}-\omega_{r}|}{\pi }\right), & \mathrm{if}\;|\omega_{g}-\omega_{r}|\;>0.1\\ 0, & \mathrm{otherwise}. \end{array}\right.$$
  where the goal point orientation is given by
  (13) $$\omega_{g}=\arctan\left(y_{goal}-y_{robot},x_{goal}-x_{robot}\right)$$

From the calculated collision penalty, goal reward, distance penalty, and heading penalty, we have the total reward function defined as:

(14) $$R=r_{c}+r_{g}+r_{d}+r_{h}$$

## 4. Training Environment and Process

The training on the navigation policy was performed on a computer system equipped with an NVIDIA GTX 1050 graphics card, 32 GB of RAM, and an Intel Core i7-6800 K CPU. The operating system used was Ubuntu 20.04 as it supports the Gazebo simulation of the Husky A200 robot. Other packages used were Tensorflow, Robotic Operating System (ROS), Python, and PyTorch.

Three training experiments were conducted to obtain three different learning policies. In each experiment, an actor–critic network (Figure 7 and Figure 8) was trained over 1000 episodes. Each episode of each experiment ends when the robot reaches its goal point, falls within the collision threshold, or reaches the timeout of 500 steps. Once the episode ends, the robot position (x,y) is set to (0.0,0.0), while the goal point and obstacles are placed randomly within the environment, ensuring that the obstacles are placed 1 m away from the goal point, as shown in Figure 6. The three experiments all use the same number of fixed-size obstacles, but varying numbers of LiDAR beams and FOV employed. The TD3 parameter update was set to two episodes while the delay rewards were updated over the last 10 steps. Also, after every 10 episodes, the network evaluates the current model in the training environment, saves the model, and records the result. Table 2 shows the description of the parameters used to train the network. The choice of the parameters was determined through experimentation and tuning.

## 5. Results

In this section, the results of the three trained models are presented. The main aim is to discuss the effect of the LiDAR beam values on the DRL algorithm. To do this, we trained the navigation policies using three different LiDAR beam values. Next, we compare the results of the three models based on their Q-values and loss values. Then, we obtain the success rate of the three models using different environments from the one used during training. Finally, we tested the effectiveness of each of the models in real-world scenarios.

### 5.1. Evaluation Metrics

Generally, the choice of metric depends on the specific goals and challenges of the task at hand to ensure that the agent is learning an effective policy. In this work, we adopt the three acceptable metrics [49,50,51]: the average Q-value, the maximum Q-value, and the loss value. During training, TensorBoard is used to visualize these metrics to determine if the agent is training well.

The average Q-value indicates the overall quality of the agent’s actions across different states. Higher average Q-values indicate that the agent is learning effective collision avoidance and path-planning strategies. The maximum Q-value provides the highest estimated return for a particular state, indicating the optimal action to take. Increasing maximum Q-values suggests that the agent is discovering more effective collision avoidance and path-planning strategies. The pseudocode for the calculation of the average Q-value and the maximum Q-value is given in Algorithm 1.
**Algorithm 1:** Average Q-value and maximum Q-value**Initialize:** Let $Q_{ij}\left(s_{t},a_{t}\right)$ be the Q-value predicted by the critic network for the state $s_{t}$ and the action $a_{t}$ during the *j*-th step of the *i*-th epoch.**Avg-Q-Step** $=\frac{1}{N.T}\sum_{i=1}^{N}\sum_{t=1}^{T}Q_{ij}\left(s_{t},a_{t}\right)$**Max-Q-Step** $=max_{i=1}^{N}max_{t-1}^{T}Q_{ij}\left(s_{t},a_{t}\right)$/* where *N* is the number of epochs, *T* is the number of steps in each epoch, and i,j,t index the epoch, step within the epoch, and time step with each episode                                                (*/

The loss value measures the difference between the predicted Q-values and the target Q-values, indicating how well the agent’s Q-value estimates align with the expected returns. The decreasing loss suggests that the agent is effectively learning collision avoidance and path-planning strategies. The pseudocode for the calculation of the maximum Q-value computation is given in Algorithm 2.
**Algorithm 2:** Loss Value**Initialize:** Let $L_{ij}$ be the critic loss during the *j*-th step of the *i*-th epoch.**Loss-Step** $=\frac{1}{N.T}\sum_{i=1}^{N}\sum_{t=1}^{T}L_{ij}$

### 5.2. Training Result

**Figure 9:**
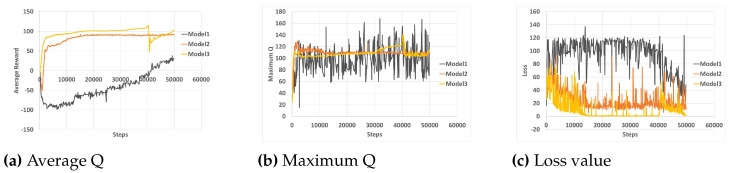
The average reward, maximum reward, and loss value for the three DRL models.

In the training analysis, we present the performance of our proposed method of obtaining a LiDAR FOV against a wider and narrower LiDAR FOV. To calculate the FOV, Equation (5) is used. The proposed model (Model 2), the narrower FOV (Model 1), and the wider FOV (Model 3) use the LiDAR configurations defined in Table 3. An increasing number of beams is used for each model because more beams within an FOV allow the sensor to capture more detailed information about the object within the limited area and can also help filter out noise clutter. Figure 9 shows the average reward, the maximum reward, and the loss values over each episode step for the three models. In the first model, where a small FOV and number of beams were considered, the average epoch value of the model starts increasing positively after training for over 30,000 episode steps with a quite high loss value over the training episodes. In the case of the other two models (Model 2 and Model 3), with a varying increase in the FOVs and number of beams, the average epoch values and the loss value progress side by side as the training progresses. However, when considering the time it took to train the models, Model 2 was the most efficient, requiring approximately 7 days to train, while Models 1 and 3 took approximately 10 and 8 days to train, respectively.

### 5.3. Simulation Performance Evaluation

**Figure 10:**
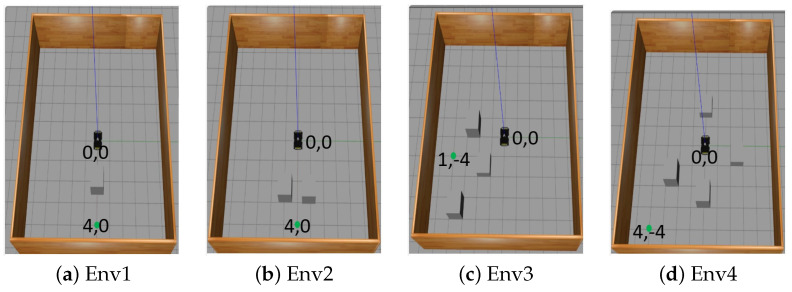
Sample of simulated environments used for testing and evaluating the three models.

**Figure 11:**
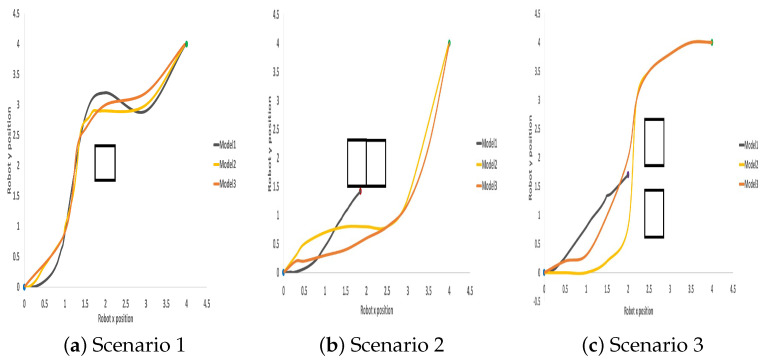
Visualization of some trajectories from the start point to the goal point of the robot for the three LiDAR-configured models; grey, orange, and gold for models 1, 2, and 3 respectively in the simulation environment. The blue marker indicates the start point, the green marker indicates the goal point, the red marker indicates collision, and the purple marker the timeout state.

**Figure 12:**
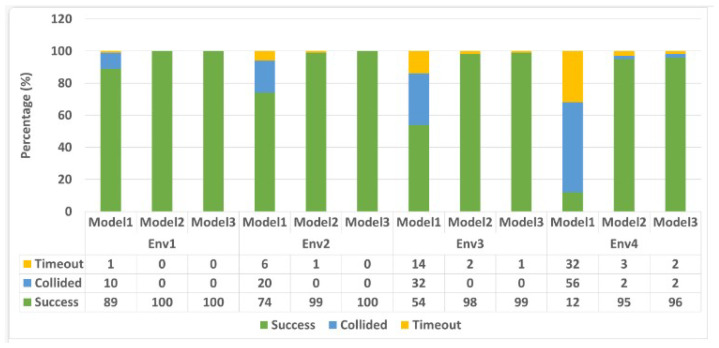
The performance of the three trained LiDAR configuration models over four testing environments. Each environment runs 100 times.

To validate the performance of the models, the models were tested in four different environment scenarios, as shown in Figure 10. In each of the scenarios, the robot is expected to reach a specified goal point 100 times. The initial position (x,y) of the robot is reset to (0.0,0.0), while for the remaining 99 tests, the robot (x,y) positions are the same as the point it was when the previous test finished except when there is a collision, in which case the robot position is set back to (0.0,0.0). The target position (x,y) is randomly generated to ensure that the target points are not placed on an obstacle and at least 1 m away from any obstacle. Figure 11 shows some of the testing trajectories, while Table 4 shows the Euclidean distance travelled and the travel time for each of the models. In these scenarios, the robot starts from the default position (0.0,0.0) to navigate to a goal point of (4,4). As shown in the results, Model 2’s travel time and the distance travelled to get to its goal point are shorter compared to the other two models. Figure 12 reports the average ratio of the success rate, collision rate, and timeout rate (when the robot did not collide or reach its goal but has exhausted the maximum number of steps it can take) for each model. These results show that the three LiDAR configuration models have a success rate of more than (85%) in the first environment scenario. However, as the dynamics of the environment become more complex, as in Env2–Env4, the success rate of model 1 falls as low as 12%. Although Model 2 and Model 3 were both able to achieve a success rate of 95%, Model 2 still performs best when the traveling time and distance traveled are considered. The LiDAR configuration used for Model 2 training outperforms the LiDAR configurations used for Models 1 and 3. Hence, the LiDAR configuration required for training a DRL in a static environment is just the angle of view to the object.

### 5.4. Real-World Performance Evaluation

**Figure 13:**
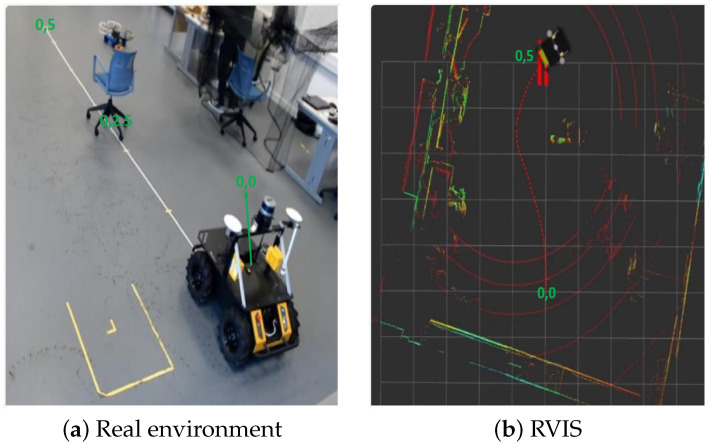
Husky robot navigation in the real-world environment. (**a**) The real-world testing environment with a static obstacle. The end of the white tape indicates the goal point. (**b**) The resultant representation of the real-world map and navigation trajectory.

Finally, to evaluate the performance of the three models, we applied the models to a physical Husky A200 robot in the real world, as shown in Figure 13. The robot has the same properties as the one used in the ROS gazebo simulation, except that the physical robot has a specific LiDAR configuration of 16 beams uniformly distributed over an FOV of 360°. The LiDAR sensor, which is mounted at the center of the top of the robot, is used to perceive the robot’s environment. The mapping and localization of the robot state are created through a wireless connection of the robot to a desktop computer. Having defined the robot’s initial position and the goal points on the map, the learning models developed were used to navigate to the goal point as shown in Figure 13.

**Figure 14:**
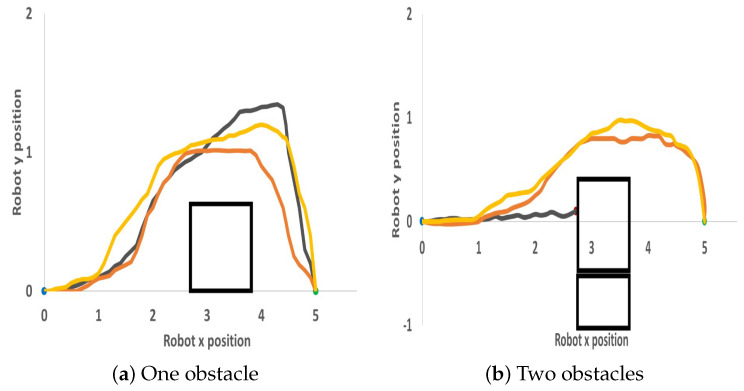
Husky robot path in the real-world environment. (**a**) Environment state with one obstacle. (**b**) Environment state with two obstacles. The grey, orange, and gold lines represent models 1, 2, and 3 respectively. The blue marker indicates the start point, the green marker indicates the goal point, and the red marker indicates collision.

The three developed models were tested in two environment scenarios. In the first environment scenario, a single obstacle was placed between the robot and the goal point, while in the second environment scenario, two obstacles were placed. As shown in Figure 14, in the first environment, the three models were able to avoid the obstacle and reach their goal point while in the second scenario, the first model collided with the obstacle, while the other models were able to avoid the obstacle.

## 6. Limitation and Future Work

While efforts were made to ensure performance quality, limitations exist in terms of verifying the performance of the proposed approach. We chose to verify the proposed method by adjusting the LiDAR configuration using the same DRL algorithm, as in the work of [24,25]. Future improvements will be to expand the study with more DRL algorithms and compare them with baseline algorithms. In terms of the dynamics of the environment used, the number of obstacles in the training and testing environment is sparse, the same size, and static. In a future study, we will improve the model by optimizing the LiDAR FOV for a clustered environment consisting of different obstacle features (size, shape, static, and moving) so that the agent can continue to learn and achieve optimal performance in the real world after deployment. Also, our performance metrics will include an analysis of the robot’s power consumption for different FOVs and the time of transmitting the control model between the robot and the CPU.

## 7. Conclusions

In this work, a study on the effects of the LiDAR configuration on the performance of the DRL for the avoidance of robot collisions based on goals has been carried out. The approach is based on using an actor–critic network to train and develop a navigation policy. Our approach considers the sensor width, a minimum safe distance between the robot, and an obstacle to generate our proposed FOV estimation. This FOV is used to configure the LiDAR sensor used during training to generate a navigation policy. The navigation policy was trained using a simulation environment with the same robot specification as in the real-life experiment; hence, they did not require any fine-tuning, allowing easy transfer of the model to the physical robot. To verify the performance of the proposed method, we adjusted the proposed FOV by reducing it by 10° to obtain a narrower FOV and another with an increased FOV of 10° to obtain a wider FOV. After comparing the three models, our proposed approach has achieved good collision avoidance of 98% success rate and an average speed of 0.25 m/s. To evaluate our approach, extensive simulation and real-world testing were performed. The experimental results show that a low-resolution LiDAR configuration can achieve high navigation performance in the context of a static environment. In future work, we intend to study the effect of varying LiDAR configurations in a dynamic environment.

## Figures and Tables

**Table 1:** Comparison between our approach and the existing LiDAR sensor configurations in the literature.

Reference	Sensor	FOV	Beam	RL-Model	Comments
[33]	LiDAR	180°	10	DDPG	The use of a small beam within a wide FOV may result in a lower point cloud density, thus impacting the level of detail.
[34]	LiDAR+Camera	180°	-	PPO	A prior knowledge of the environment is required.
[35]	LiDAR	270°	512	PID+CNN	The model performs well, however, this choice of LiDAR configuration for such a task seems costly.
[36]	LiDAR	13.34°	Learns	ε-Greedy Search	The optimization was specific to just the beam size and not the FOV. The method performs well for localization and object detection.
[37]	LiDAR	Varies	Varies	SAC	Does it mean that 240° FOV is the best for all types of environments or scenarios?
[38]	LiDAR	25°	72	A3C	A sensor with more beams affects its mechanical design and is more costly.
[39]	Camera	90°	18	LSTM-LMC	The camera data need to be processed into point cloud data.
Our Approach	LiDAR	$\theta =2*\arctan\frac{h}{2*L}$	20	TD3-AC	The required FOV is calculated based on the width of the LiDAR *h* and the minimum distance *L* set between the obstacle and the robot.

**Table 2:** Learning parameters for the TD3 network.

Learning Rate	Discount Factor	Update Rate	Policy Noise	Batch Size
0.0005	0.99999	0.005	2	200

**Table 3:** LiDAR configuration settings.

	Model 1	Model 2	Model 3
FOV	θ−ϕ	θ	θ+ϕ
Number of beams	10	20	30
		N.B: *ϕ* = 10°	

**Table 4:** Test travel time and Euclidean distance traveled.

	Models	Scenario 1			Scenario 2			Scenario 3		
Criteria		1	2	3	1	2	3	1	2	3
Distance (m)		2.79	**1.81**	2.39	Timeout	**2.79**	3.40	Collision	**1.83**	2.63
Time (s)		9.52	**8.05**	8.92	Timeout	**9.5**	10.44	Collision	**8.08**	9.28

## Data Availability

A video illustrating the real-world experiment can be seen at https://youtu.be/EghnBnaeDeM (accessed on 2 October 2023) and the code is publicly available at https://github.com/kabirat/DRL_LiDAR_Config (accessed on 25 September 2023).
